# Integrase-Controlled Excision of Metal-Resistance Genomic Islands in *Acinetobacter baumannii*

**DOI:** 10.3390/genes9070366

**Published:** 2018-07-20

**Authors:** Zaaima AL-Jabri, Roxana Zamudio, Eva Horvath-Papp, Joseph D. Ralph, Zakariya AL-Muharrami, Kumar Rajakumar, Marco R. Oggioni

**Affiliations:** 1 Department of Genetics and Genome Biology, University of Leicester, Leicester LE1 7RH, UK; zaljabri01@gmail.com (Z.A.-J.); rzz1@leicester.ac.uk (R.Z.); jdwr2@leicester.ac.uk (J.D.R.); 2 Department of Microbiology and Immunology, Sultan Qaboos University, Muscat 123, Oman; muharrmi@squ.edu.om; 3 Department of Infection Immunity and Inflammation, University of Leicester, Leicester LE1 7RH, UK; ehp5@student.le.ac.uk (E.H.-P.); kr46@le.ac.uk (K.R.)

**Keywords:** copper resistance, genomic island, integrase, *Acinetobacter baumannii*, mobile genetic element

## Abstract

Genomic islands (GIs) are discrete gene clusters encoding for a variety of functions including antibiotic and heavy metal resistance, some of which are tightly associated to lineages of the core genome phylogenetic tree. We have investigated the functions of two distinct integrase genes in the mobilization of two metal resistant GIs, G08 and G62, of *Acinetobacter baumannii*. Real-time PCR demonstrated integrase-dependent GI excision, utilizing isopropyl β-d-1-thiogalactopyranoside IPTG-inducible integrase genes in plasmid-based mini-GIs in *Escherichia coli*. In *A. baumannii*, integrase-dependent excision of the original chromosomal GIs could be observed after mitomycin C induction. In both *E. coli* plasmids and *A. baumannii* chromosome, the rate of excision and circularization was found to be dependent on the expression level of the integrases. Susceptibility testing in *A. baumannii* strain ATCC 17978, A424, and their respective ΔG62 and ΔG08 mutants confirmed the contribution of the GI-encoded efflux transporters to heavy metal decreased susceptibility. In summary, the data evidenced the functionality of two integrases in the excision and circularization of the two *Acinetobacter* heavy-metal resistance GIs, G08 and G62, in *E. coli*, as well as when chromosomally located in their natural host. These recombination events occur at different frequencies resulting in genome plasticity and may participate in the spread of resistance determinants in *A. baumannii*.

## 1. Introduction

Genomic islands (GIs) are discrete gene clusters most of which are found as DNA segments within the chromosome. The GIs were originally known as pathogenicity islands (PAIs) by Hacker et al. in late 1980s, when they were examining the genetic virulence mechanisms in *Escherichia coli* [1]. Genomic islands are variable in size, ranging from 10 to 200 kb, and are usually detected during comparative genomic analysis of different closely related strains [2]. Genomic islands generally harbor genes coding for an integrase or recombinase, but could also carry insertion sequences or transposons within the element contributing to their movement [3,4]. Genomic islands are by definition part of the accessory gene pool of a species and allow for mobilization of whole pathways and gene clusters, thus acting as reservoirs for genetic diversity [5].

In the last few decades, *Acinetobacter baumannii* has been recognized as a multi-drug resistant opportunistic pathogen as a result of being burdened with massive use of broad-spectrum antibiotics [6]. The presence of *A. baumannii* with closely related Gram-negative bacteria in clinical settings has contributed to the development of new resistance mechanisms on top of their own intrinsic factors [7]. *Acinetobacter baumannii* and the other Gram-negative pathogens are known for their genome plasticity and capability of evolving mainly due to acquiring new virulence determinants carried on mobile genetic elements [8]. This issue has rendered antibiotic therapy ineffective in life-threatening *A. baumannii* infections, and in some cases even with the last line of combination therapy of high doses of antibiotics [9].

In *A. baumannii,* the first GI identified was a large GI of 86 kbp in size, during the sequencing of the epidemic strain AYE, and was named “AbaR1” harboring 45 resistance determinants [10]. Similar GIs were later recognized in *A. baumannii* and named from AbaR0 to AbaR27 [10,11,12]. Despite being similar in the backbone structure, AbaR-like GIs are often variable in terms of size and genetic composition. For example, the multiple-antibiotic resistance region (MARR) of the AbaR GI harbor a set of genes encoding antibiotic, heavy metal, and antiseptic resistance and efflux determinants [10]. This diversity in AbaR GIs compositions were a result of several events of recombination like integration, excision, and rearrangements [13].

Comparative genomic analysis showed extensive synteny throughout the genome and identified 63 DNA regions, ranging in size from 4–126 kb, all exhibiting certain features of GIs including a group of resistance GIs with different genes encoding resistance to antibiotics and heavy metals which are grouped in clusters [14]. For example, the *aadA1* (streptomycin-resistance encoding) gene, flanked by *satR* (streptothricin-resistance encoding) and *dhfr* (trimethoprim-encoding resistance) genes were found in GIs in clusters. Moreover, genes involved in mercury resistance (*merRCAD* cluster) were found to be located in a separate cluster, and a 4.5 kb DNA segment containing *feoAB* (ferrous iron transport operon), *czc* (tricomponent proton/cation antiporter efflux system), and *ars* (arsenite transporters) genes were co-existing as a group, next to the *cus* (copper resistance) genes conserved in the same chromosomal locations of certain GIs [14]. However, these genes differ in sequence and the overall arrangement from other homologous GIs in *A. baumannii*. This supports the notion that the set of accessory genes had been independently acquired by the different strains.

**Figure 1:**
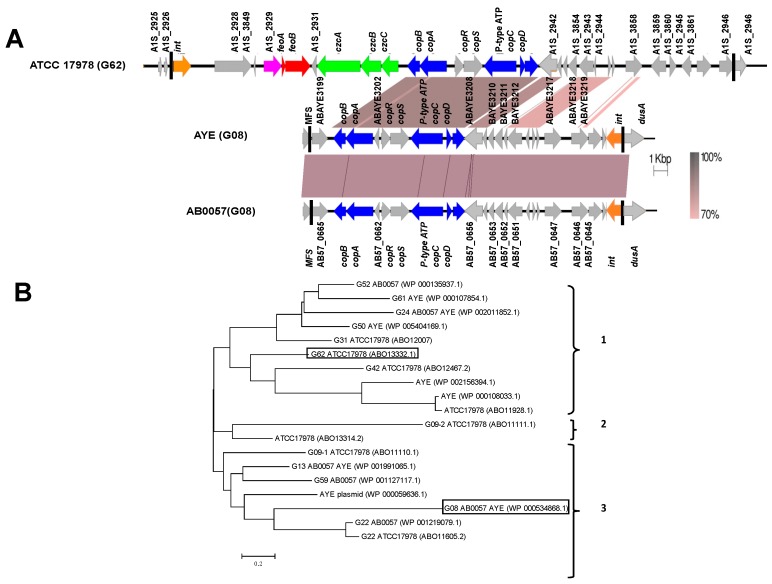
Schematic map of the *Acinetobacter baumannii* strain genomic islands G08 and G62 and phylogenetic tree of genomic island (GI) integrases. (**A**) G62 island is taken from strain ATCC 17978 (accession CP000521; updated refseq NC_009085.1) and G08 from both strains AB0057 (NC_011586.2/CP001182.2) and AYE (NC_010410.1/CU459141.1). The annotated sequences were aligned and visualized by the Easyfig tool [21]. The genes involved in encoding copper efflux systems are shown in blue, all *czc*-like genes *czcA*, *czcB*, and *czcC* encoding cadmium, zinc, and cobalt resistance are represented in green color, genes encoding ferrous iron transport proteins are shown in red (*feoA* and *feoB*), and a putative further heavy metal efflux system A1S_2929 is shown in pink. The integration sites (*att* sites) for both GIs are shown as black vertical lines. The genes encoding the integrases are colored in orange and all other genes in grey. Locus tags of relevant genes are shown above or below the genes. The image is drawn in scale and the percentage of DNA identity between various regions is shown by gradient shading. (**B**) Phylogenetic tree of tyrosine recombinases from strains ATCC 17978, AB0057, and AYE. The evolutionary relationship between phage integrase family proteins (NCBI Reference Sequence) detected in three strains of *A. baumannii* was inferred using the Neighbor-Joining method [22]. Integrases were labelled according to the genomic island number, strain name, followed by Refseq accession numbers. In certain instances, some GIs have not been given a specific number, but instead were indicated by their accession. When two strains are mentioned next to a GI, this means that the same GI is present in both strains. Identical proteins in different genomes which yield identical Refseq numbers are shown only once. The three major branches are indicated by numbers on the right side of the figure. G08 and G62 are indicated by black boxes.

The two GIs of interest in this study, G08 and G62, harbor a set of putative heavy metal resistance conferring genes which are identical (Figure 1A) [14]. Only a few studies have described the G62 island [14,15,16], for example the presence of a similar resistance island has been shown in an *A. baumannii* hyper-virulent and outbreak-associated isolate, LAC-4 in China [15]. In LAC-4 clinical isolate, the G62 harbors the exact set of resistance genes and was referred to as a “copper resistance gene cluster”; however, in that strain, G62 was found to be sandwiched between two copies of IS*Aba26* element [15]. The ATCC17978 genome has been extensively analyzed in previous studies [17] and 13 putative zinc/copper resistance efflux pumps have been identified, including the efflux pumps present in G08 and G62 [16] (Figure 1A). The chromosomal region harboring zinc and/or copper efflux genes are likely to have been acquired laterally on mobile genetic elements, with the G62 of ATCC 17978 and LAC-4 being the largest of these elements [16]. Comparative analysis of putative zinc and/or copper efflux systems in *A. baumannii* and *A. baylyi* (strain ADP1) other than the ones in G08 and G62, identified a number of genes ranging between eight (strain SDF) and 18 (strain AB6870155) in each of the strains examined, all of which were chromosomally located [14,18]. Further BLAST search revealed that five strains harbored more than ten genes encoding putative zinc and/or copper efflux components, including ATCC 17978, ATCC 19606T, AB0057, AB6870155, and ACICU. On the other hand, the G08 island is found more frequently in *A. baumannii* including in strains AB0057, AB6870155, and AYE, where the element is inserted into the *dusA* locus encoding for the enzyme tRNA-dihydrouridinesynthase A, catalyzing the post-transcriptional reduction of uridine to dihydrouridine in tRNA [19].

The analysis of the distribution of genomic islands and other genes belonging to the accessory genome has shown in many species that many so-called mobile elements or accessory genes cluster tightly with specific lineages on a phylogenetic core genome tree [5,10,20]. This raises questions on this association which could be due to some positive selection or more likely due to loss of function of the mobile elements. In this work, we aim to test the hypothesis that the two metal-resistant related genomic islands G08 and G62 of *A. baumannii* are still functional.

## 2. Materials and Methods

### 2.1. Bacterial Strains and Cultivation

A set of *A. baumannii* strains from different geographical origins were used. Hundred strains were collected from clinical samples at Sultan Qaboos University Hospital (SQUH, Oman) between 2012 to 2013. These strains were collected from various body sites of patients admitted in the internal medical wards in SQUH. The rest of the strains were from the collection at the Department of Infection, Immunity and Inflammation of the University of Leicester (Table 1). All strains were stored at −80 °C in 30% glycerol. Strains were re-streaked in LB agar (BD) or broth for liquid cultures.

### 2.2. Genome Analysis

The whole genome phylogenetic single nucleotide polymorphism (SNP) tree was build using the FFP (Feature frequency profile) version 3.19 suite of programs (http://sourceforge.net/projects/ffp-phylogeny/) [25]. As input, the 101 complete *A. baumannii* genomes deposited in GenBank (8 July 2018) were used with the addition of our own two strains A424 (GCA_003185755.1) and KR3831 (GCF_003185745.1/GCA_003185745.1). The matrix of integrase presence in the genomes was generated using as a query the integrase genes present in AB0057, AYE, and ATCC 17978 (shown also in Figure 1B). The matrix was generated using command line BLAST (90% identity; 95% coverage) and the R platform to generate the output.

### 2.3. Colony Genotpying

The polymerase chain reaction was performed on crude cell extracts from serially diluted suspensions of *E. coli* and *A. baumannii* cells. Thirty µL aliquot of the dilution was boiled for 5 min, and 2 µL of supernatant was used as a polymerase chain reaction (PCR) template. PCR was conducted in a 20 µL reaction volume, containing 1 µL DNA (50 ng/µL), 5 µL 10× reaction buffer (Promega, Madison, WI, USA), 1 µL dNTPs (10 mM), 1 µL of each 10 mM primer, and 0.2 µL Go*Taq* DNA polymerase (Promega). Amplification was performed in a thermal cycler (Mastercycler gradient, Eppendorf, Hamburg, Germany) with an initial denaturation at 95 °C for 2 min, followed by 25 cycles of 95 °C for 1 min, 57 °C for 1 min, and 72 °C for 2 min, and a final extension at 72 °C for 10 min. The PCR products were imaged from a 1% TAE-agarose gel.

### 2.4. Real-Time PCR

The real-time PCR reactions had a total volume of 20 µL containing 5 µL of template DNA, 10 µL of the SensiMixPlus SYBR Green mastermix (Bioline, London, UK), and 0.5 µL of each 15 µM primer (F-G08-exc and R-G08-circ) or (F-G62-exc and R-G62-circ). Since there was no positive control used, every run included a negative control without target DNA, and all reactions were performed in triplicate. The reactions were performed in an Applied Biosystems Prism (Foster City, CA, USA) model 7500HT Sequence Detection System with the following settings: 40 cycles of 20 s at 95 °C and 1 min at 60 °C. Determinations of cycle threshold (Ct), or the PCR cycle where fluorescence first occurred, were performed automatically by the Sequence Detection Systems software of the instrument (version 2.3; Applied Biosystems, Foster City, CA, USA).

### 2.5. RNA Extraction and Retrotranscription

Following induction, 5 mL of cells were harvested at predetermined time points by centrifugation for 5 min at 4000× *g*, and resuspended in 1 mL RNALater (Invitrogen, Carlsbad, CA, USA), and stored at 4 °C. The total RNA was extracted using the Geneflow total RNA purification kit protocol (Norgen, Thorold, ON, Canada). The RNA was eluted from the column into a 1.5 mL microcentrifuge tube by addition of 30 μL RNase-free water and stored at −20 °C. Total RNA was quantified by spectrophotometry at A260 (Nanodrop 2000; Fisher ThermoScientific, Waltham, MA, USA), and cDNA was created by taking 1 μg of RNA per each reverse transcription reaction which was 20 μL, and the procedure was completed according to the High Capacity RNA-to-cDNA kit (Applied Biosystems).

### 2.6. Construction of Plasmids with G08 and G62 Mini-GIs

To test the functionality of the integrase genes of G08 and G62 in excising their respective GIs, two plasmid constructs were created. Mini-islands were generated by creating smaller circular molecules with precise site-specific excision via the attachment sites *attL*/*attR* included within the left and right flanking regions, and integrase coding gene cloned in a plasmid under an inducible promoter. The new fragments generated were later cloned into pUC18 vector. The primer pairs F-LF-08/R-LF-08 and F-RF-08/R-RF-08 (Table S1) were used to amplify the left and right G08 flanking regions including the *att* sites from strain A424 (Genbank accession: GCA_003185755.1). Similarly, the left- and right-flanking regions including the *att* sites of G62 GI were amplified from the strain ATCC 17978 using the primer pairs F-LF-62/R-LF-62 and F-RF-62 and R-RF-62 (Table S1), respectively. The two integrase genes G08*int* (A424_1287 from A424) and G62*int* (A1S-2927 from ATCC 17978) were separately amplified by PCR using primer pairs F-G08int/R-G08int and F-G62int/R-G62int, respectively. Amplicons containing the integrases were ligated in the HindIII within the multiple cloning site (MCS) to be expressed under the *lacZ* promoter in the final recipient vector. The three PCR fragments (LF, RF, and integrase) were finally joined by fusion PCR resulting in a recombinant DNA product.

### 2.7. Construction of Inducible Plasmids for A. baumannii

The vectors carrying the min-islands of G08 and G62 were sub-cloned into pWSK129, a low- copy-number plasmid carrying aminoglycoside 3′-phosphotransferase (*aphA1*) gene conferring kanamycin-resistance (Km^R^) [26]. As this plasmid turned out to be non-functional in *A. baumannii*, we amplified the origin of transfer from pWH1277, a cryptic plasmid from an *A. lwoffii* strain fragment of pWH1266 (kindly donated by Philip Rather, Emory University, USA), using the primer pair PR3136 and PR3137 (Table S1). These pWSK129-WH plasmids were successfully transferred into competent *A. baumannii* knock-out strains (A424 and ATCC 17978) by conjugation.

### 2.8. Suicide Vector-Based Allelic Exchange for Mutant Construction in A. baumannii

Deletion mutants of the GIs were constructed in *A. baumannii* using the suicide vector pJTOOL-3 [27], containing 500 bp long fragments of each of the borders of either G08 or G62. For transformation, *E. coli* CC118λ*pir* and S17.1λ*pir* were used as a host for replication and as a conjugative strain, respectively. Plasmid single cross-over insertion into *Acinetobacter* was selected by gentamicin and the double cross over by plating on 6% sucrose containing to check for the loss of the levansucrase *sacB* gene of pJTOOL-3. The expected genotype was obtained in all three randomly selected colonies that possessed the expected chloramphenicol-sensitive and gentamicin-resistant phenotype.

### 2.9. IPTG and Mitomycin C Induction

For isopropyl β-d-1-thiogalactopyranoside (IPTG), 5 mL overnight culture of *A. baumannii* or *E. coli* were diluted at 1:100 into fresh LB and then incubated at 37 °C in the shaking incubator at 200× *g*, until OD_600nm_ = 0.2 is reached. IPTG was added at concentration of 1.0 mM, and the cultures were then incubated, with 500 μL of the culture removed at time points 0, 4, 8, and 24 h for DNA preparation and qPCR analysis. In *Staphylococcus*, mitomycin was shown to induce excision of genomic islands [28]. For mitomycin C, induction 5 mL of overnight cultures of *A. baumannii* strains were treated with sub-lethal concentrations of mitomycin C MIC (0.5 times the MIC) for 2 h. Mitomycin C MIC of AYE, AB0057, A424, KR3831, and ATCC 17978 was found to range from 32–64 µg/mL. Non-induced cultures were run alongside in each occasion under identical conditions.

### 2.10. Metal Susceptibility Testing

For testing of susceptibility to the heavy-metal salts, analytical-grade salts of CdCI_2_·H_2_O, CoCl_2_·6H_2_O, NiSO_4_·6H_2_O, and ZnSO_4_·7H_2_O, CuSO_4_·5H_2_O, FeSO_4_·7H_2_O, MnSO_4_·H_2_O. and As_2_SO_3_ (Sigma–Aldrich, Gillingham, UK) were used to prepare 1.0 M stock solutions, which were dissolved in ultrapure distilled water and later filter-sterilized and added to the medium at final concentrations of 1 mM. MIC and MBC assays to heavy metals was performed as described by the Clinical and Laboratory Standards Institute (CLSI) guidelines using a broth microdilution method [29]. Briefly, starting inocula of 1 × 10^5^ CFU/mL of all *A. baumannii* strains were aliquoted in 96-well plates containing serial dilutions of each metal compound in the range 0.02–10 mM using MHB (Oxoid Ltd., Basingstoke, UK).

## 3. Results

**Figure 2:**
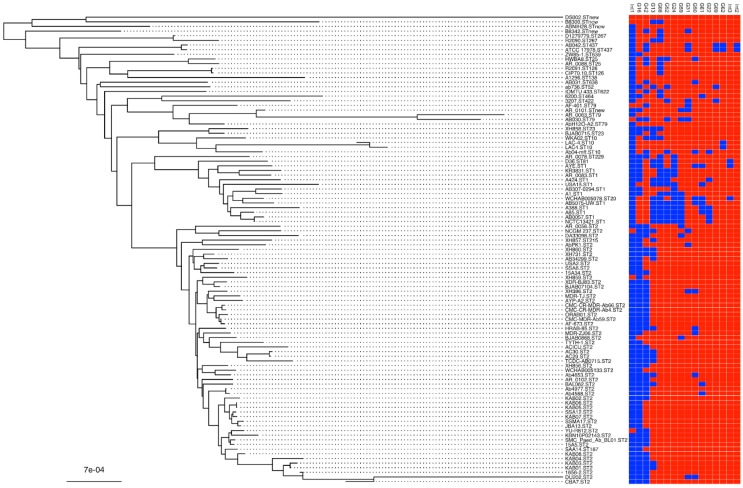
Integrase distribution on an *A. baumannii* phylogenetic tree. The whole genome phylogenetic SNP tree was built using the FFP and input from the 105 complete *A. baumannii* genomes deposited in GenBank (08/07/2018) with the addition of strains A424 and KR3831. Strain names include the MLST “Pasteur” sequence type. The matrix of 16 integrases on the right part of the figure includes all integrase genes present in AB0057, AYE, and ATCC 17978 (red absent; blue present). The integrases of the GI are numbered according to Di Nocera et al. [14]. Three further integrases present in the three stains but not associated to genomic islands were included in the analysis and named *int1* (ABAYE_RS10930), *int2* (AUO97_RS03560), and the p3ABAYE integrase *int3* (ABAYE_RS00155).

The distribution of integrases, as proxies of their genomic islands, varies widely in different lineages of a whole genome phylogenetic SNP tree constructed on all complete deposited *A. baumannii* genomes (Figure 2). One of the integrases not associated to genomic islands (*int1* ABAYE_RS10930) present in almost all isolates, some others such as G08, G13, G16, and G42 are detected in many lineages and may be present only in a subgroup of isolates of a given ST. Other integrases like G09, G31 or G62 are present only in single or very few STs. The integrase of the metal resistance associated genomic islands G08 and G62 are representatives of this latter groups being G08 present in ST1, 25, 26, 52, 79, 81, 126, 138, 229, 422, and 638m while G62 only in ST10 and 437 (Figure 2).

In order to test the hypothesis that these islands, even if present only in defined lineages are still mobile, we selected the genomic islands G08 and G62 respectively in strains AB0057 and ATCC 17978 [14] (Figure 1A). Phylogenetic analysis of the respective GI integrases confirmed that the G08*int* and G62*int* are not related, and each belong to a separate clade within the *Acinetobacter* GI-related tyrosine recombinases (Figure 1B). Both GIs carry the *copABCD* and *copRS* copper resistance genes [16], and G62 carries in addition the cadmium, zinc, iron, and cobalt resistance genes (Figure 1A). To check the distribution of G08 and G62, we screened by PCR a 100 sample collection of *A. baumannii* clinical isolates obtained from SQUH, Oman from 2012 to 2013 using primers for conserved sequences flanking the target region. The PCR screening analysis yielded a possibly occupied G08 in only one clinical isolate, and the presence of G08 was confirmed by WGS (strain KR3831, accession GCF_003185745.1, GCA_003185745.1). To test the contribution of G08 and G62 to metal susceptibility phenotypes, we constructed deletion mutants respectively in strain A424 and ATCC 17978 (Table 2). In the G08 knock out mutant, only the minimal inhibitory concentration (MIC) for manganese (MnSO_4_) decreased form 1 μg/mL to 0.5 μg/mL, while the copper MIC remained unchanged. Deletion of G62 in ATCC 17978 resulted in a decrease of the MIC of zinc (ZnSO_4_ from 4 to 2 μg/mL), cobalt (CoCl_2_ 4 to 2 μg/mL), cadmium (CdCl_2_ 4 to 2 μg/mL), and nickel (NiSO_4_ 4 to 2 μg/mL), and again no decrease in the MIC of copper was detected (CuSO_4_ 8 μg/mL). No differences were observed in susceptibility to iron (FeSO_4_) and arsenic (As_2_O_3_). Four independent ko mutants were assayed against the wild type in all tests and the difference in MICs found to be statically relevant.

**Figure 3:**
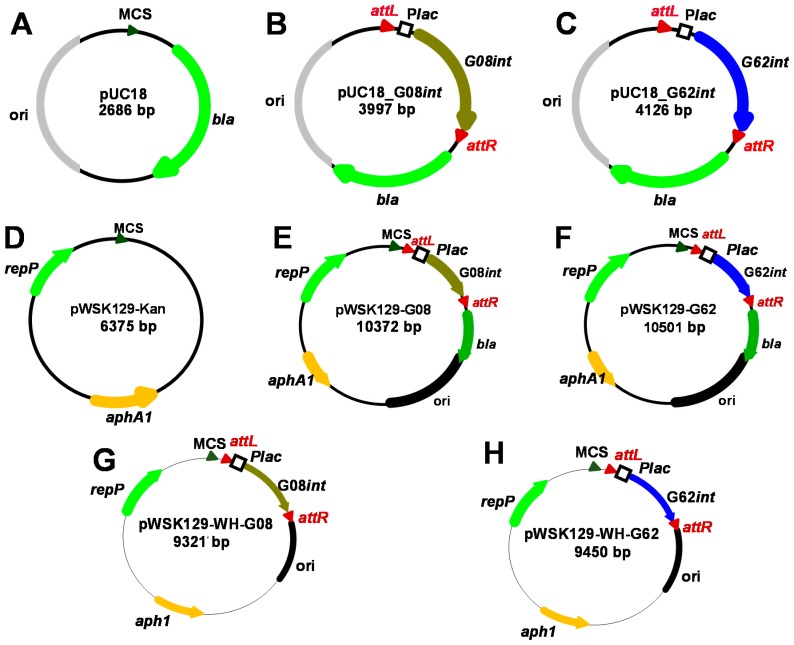
G08 and G62 mini-GI vectors for *E. coli* and *A. baumannii*. pUC18 (**A**) used as a vector harboring *bla* gene conferring ampicillin resistance (green), *E. coli* origin of replication *ori* (grey), multiple cloning site MCS shown as green triangle, to clone mini-GIs containing upstream and downstream flanking borders with *attL/attR* sites (red) of the respective islands as well as integrase genes (**B**) G08*int* (olive) and (**C**) G62*int* (blue). The IPTG-inducible P*lac* promoter was fused by PCR in front of the integrase genes during construction of the plasmids (**B**,**C**). Plasmids used to assess integrase activity in *A. baumannii* (**D**–**F**) were constructed by fusing pUC18-based G08 and G62 mini-island vectors with pWSK129 carrying the *aphA1* conferring kanamycin resistance (orange) and pLG339 replication initiation protein (*repP*, light green). To allow for stable transfer of the constructs to *Acinetobacter* pWSK129-WH-G08 (**G**) and pWSK129-WH-G62 (**H**) were constructed. pWSK129-kan plasmids were used to clone the *Acinetobacter* origin of Replication (*oriR*) from pWH1266 (black color) resulting in pWSK129-WH-derived new constructs compatible with *A. baumannii* strains.

**Figure 4:**
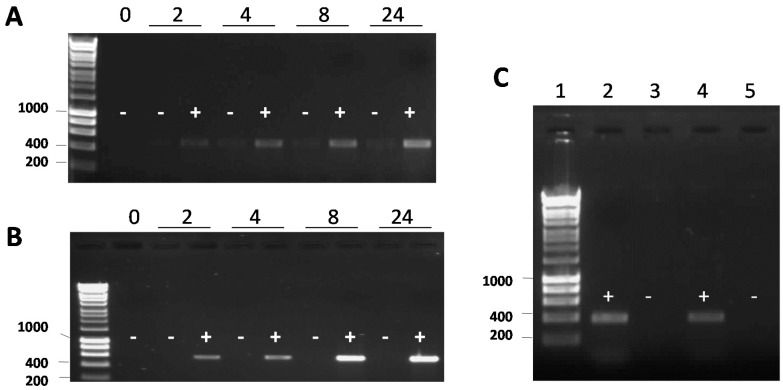
Integrase induction drives excision of G08 and G62 mini-islands in *E. coli* and *A. baumannii*. Gel images of circularized G08 (from AB0057) (**A**) and G62 (from ATCC 17978) (**B**) mini-islands respectively, with (+) and without (−) IPTG induction. Mini-GI excision was tested by amplification of circular intermediates using outwards facing primer sets yielding 567 bp product for G08 (**A**) and a 489 bp product for G62 excision (**B**). Different time points (in hours) after IPTG induction in early exponential phase are shown (legend above gel). Integrase dependent excision of G08 and G62 mini-islands in *A. baumannii* is shown in panel C. Gel images of circularized G08 and G62 mini-islands 8 h after IPTG induction with the same primers as in *E. coli* (panel **A** and **B**). The non-induced samples in lane 3 for G8 and in lane 5 for G62 in lanes 4 and 5 of panel C. The marker is Gene Ruler 1 kb and the size of some bands in bp is given on the left of each gel.

To test the functionality of integrase genes of the G08 and G62 islands in excising their respective GIs in a heterologous *E. coli* background, mini-islands were generated. Mini-GIs were obtained by cloning the integrase coding genes G08*int* (locus_tag A424_1287, strain A424, NC_011586.2/CP001182.2) and G62*int* (locus_tag A1S-2927, strain ATCC 17978, NC_009085.1/CP000521) under control of the *lacZ* promoter and flanked by *attL* and *attR* sites [30] (Figure 3). The resultant plasmids pUC18-G08*int* and pUC18-G62*int* carrying the mini-islands of G08 and G62 were complemented in their respective knock out strains to be tested for integrase dependent excision using sets of divergent primers. The *dusA*–associated integrases have been shown to excise as circular elements with the restoration of the junction [19]. Our data showed IPTG-dependent mini-GI excision, by amplification of both the reconstituted target site and the junction of the circular form, for both the G08 and G62 constructs over the whole growth phase in liquid medium (Figure 4A,B). No or only marginal excision of the mini-GIs was detected without IPTG induction of the plasmid-carried mini-GIs (Figure 4A,B). To test for the excision of mini-GIs in *A. baumannii* background, the constructs were transferred on pWH1266 and pWK129 shuttle vectors. The IPTG-induced excision of the mini-GIs in *A. baumannii* was detected by amplification of the circular intermediates of the G08 and G62 mini- GIs using the same primer sets as in *E. coli* (Figure 4C).

**Figure 5:**
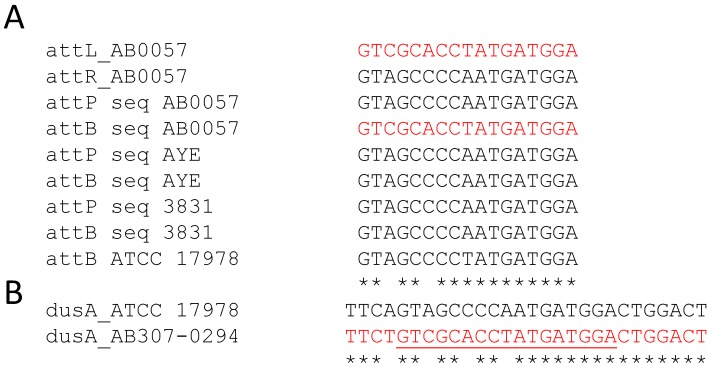
Allelic variation in the *attB* site of *A. baumannii* strains. (**A**) Shows the variability of *att* sequences in AB0057 and AYE and KR3831 compared to ATCC 17978. The *att* sites sequenced in this study are abbreviated as (seq). (**B**) Shows two variable alleles of *attB* sequences in *dusA* in two *A. baumannii* strains ATCC 17978 and AB307-0294 lacking the G08 island. The *attB* site is underlined. The variable alleles differing from the consensus sequence by two or three SNPs are shown in red.

To test the dynamics of excision and reconstitution of the chromosomal target site of the G08 and G62 islands in *Acinetobacter*, we amplified the circular intermediates and targets in our four G08-positive AB0057, AYE, A424, and KR3831 strains, as well as the G62-positive strain ATCC 17978. To test GI excision, bacteria were grown to mid log phase and either tested directly or after exposure to 38 µg/mL of mitomycin C for 2 h. The junction and the circular forms were sequenced by Sanger sequencing to map the *att* sites of G08 and G62. In ATCC 17978, the *att* sites of G62 were identical at both ends with the consensus “AATAACTTTAAAGATTAA” [14]. However, our data show that in all examined strains, G08 was flanked by two 17 bp semiconserved attachment sequences [19], which showed variation in the *attR/attL* and *attP/attB* of a single nucleotide (SNP) in strains AYE and KR3831, and of two SNPs in strain AB0057 compared to ATCC 17978 (Figure 5). To examine the reason for these differences in the *att* sites among G08-harbouring strains, the database was searched for *att* sites of strains devoid of G08 and showed that two variable alleles of the *attB* sites in *dusA* gene exist in two G08-negative strains ATCC 17978 and AB307-0294. The ATCC 17978 had the more frequently occurring allele with a single SNP, whereas AB307-0294 had two SNPs similar to those seen in AB0057. This could mean that either of the *att* sites could be recognized by the integrases as preferable integration/excision sites during mobilization of the G08.

**Figure 6:**
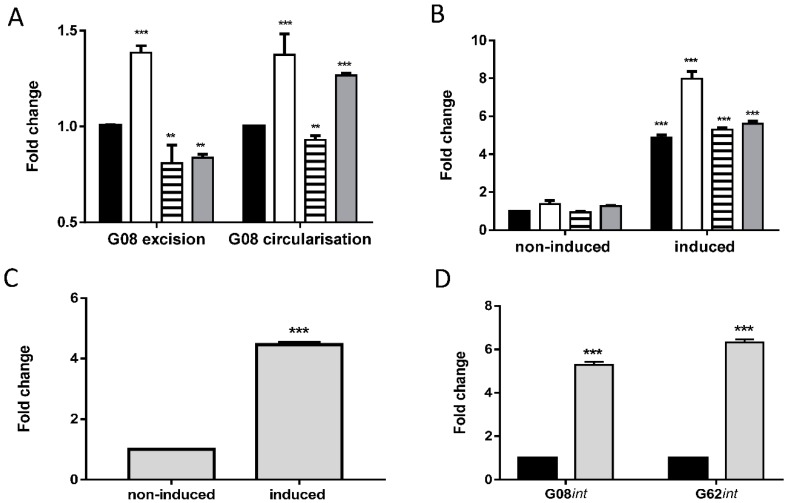
Quantification of G08 and G62 island excision and integrase expression in *A. baumannii* strains with and without mitomycin C induction. The data in (**A**) represent the real-time PCR detection of reconstituted target sites after excision of G08 (excision) and of the circular intermediates (circularization) without any induction. The G08 positive strains are AB0057 strain (black, accession NC_011586.2/CP001182.2). AYE (white, accession NC_010410.1/CU459141.1), A424 (striped, accession GCA_003185755.1), KR3831 (grey, accession: GCF_003185745.1. GCA_003185745.1), and data are normalized to AB0057. (**B**) Reports the variation in G08 excision by detecting circular intermediates with and without mitomycin C induction (0.75 × MIC) for 2 h. The asterisks represent the significance of change in each strain when compared to AB0057. Error bars represent SEM of three independent replicates. (**C**) Repost real-time PCR quantification of G62 circular intermediates with and without mitomycin C induction. (**D**) Shows the expression G08*int* (A424_1287 from strain A424, protein ID: PRJNA473420:DMB35_05960, Genbank accession: GCA_003185755.1) and G62*int* (A1S-2927 from ATCC 17978) measured by real-time PCR without or with mitomycin C exposure (grey). Error bars indicate the SEM of three independent replicates in each experiment as analyzed by two-way ANOVA test. ** *p* < 0.01, *** *p* < 0.001.

Real-time PCR was performed to quantify the circular forms after excision and variation in number of excised elements between samples was corrected by arbitrarily setting the values of strain AB0057 to 1 and expressing the data in the other strains and after mitomycin C treatment at fold change. The primers’ efficiencies were checked by performing serial dilutions (Figure S1). Without any induction, the excision of the elements was low and no strong baseline variation between the G08 carrying strains AB0057, AYE, A424, and KR3831 were seen for both the detection of the reconstituted target site and the circular intermediates (Figure 6A). After exposure of *A. baumannii* cells to mitomycin C, the detection of G08 circular intermediates increased in all strains significantly 4- to 8-fold (Figure 6B). Similarly, when testing excision of the G62 element in strain ATCC 17978, we detected a significant increase of about 4-fold in the formation of circular intermediates after exposure to mitomycin C (Figure 6C).

To test whether the increased excision of the G08 and G62 elements after mitomycin C treatment in *A. baumannii* was integrase mediated, we tested the expression of the integrases G08*int* (A424_1287 from A424) and G62*int* (A1S-2927 from ATCC 17978). This was done by real-time PCR with primers internally to G08*int* and G62*int*. Data show significant upregulation of integrase expression after mitomycin C exposure of about 5-fold for G08*int* and 6-fold for G62*int* (Figure 6D).

## 4. Discussion

Previous comparative genomic analysis of *A. baumannii* explored the chromosomal loci of 63 GIs including the two GIs objects of this study, G08 and G62, within seven strains belonging to different genotypes ST1, ST2, ST25, ST77, and ST78 [14]. The genomic alignment revealed GIs of various functions such as those encoding for surface components and transport systems, as well as resistance to drugs and heavy metals. More recently, data of a pan-genome analysis of 50 *A. baumannii* isolates and 249 previously sequenced *A. baumannii* strains were compiled [31], and their dataset confirmed the diversity of gene pools found within the GIs identified as an adaptive response of the *A. baumannii* strains to facilitate their survival in a nutrient-deficient environment. In many instances, the integrases of these GIs were found to be non-functional in various species due to frameshift and nonsense-mutations [32,33,34]. This resulted in most of these GIs being permanently positioned in their chromosomal location [35]. Therefore, this work aimed to check whether the G08 and G62 integrases are functional and contribute to excision of the islands by generating mini-GIs carrying the essential components for mobilization of integrases and *att* sites. In addition, direct excision from the chromosomal host was tested via mitomycin C induction. This approach has been previously employed in other *A. baumannii* studies [36] as well as other GIs circularization and have successfully demonstrated excision after the use of modified protocols [37,38,39]. The response to mitomycin C that was studied and research showed that pathogenicity island excision was facilitated by mitomycin C which induces an SOS response [28,40]. The data presented here have confirmed that the use of mitomycin C can effectively induce the excision of the GIs G08 and G62 via the visualization of the bands in gel electrophoresis. Similar observations were reported on other *dusA/dusB* associated integrases [19]. Real-time PCR data supported our observation and even demonstrated excision and circular events occurring at later cycles without induction. Sequencing of the circular intermediates and the chromosomal junctions after excision showed the possibility of having multiple *att* sites in AB0057, which probably can lead to having multiple insertions occurring at different frequencies depending on the most prevalent or preferable sites.

It could also be argued that the importance of such variability in attachment sites is probably minor, due to the low excision frequencies under laboratory conditions. Studies in which integrase activity was assessed in *A. baumannii* background are limited, most of which were performed in integron studies in the closely related non-pathogenic species *Acinetobacter baylyi* due to the ease of genetic modification and transformations [41,42,43]. In this context, our study demonstrated integrase-dependent excision and circularization in both tested metal-resistance GIs that were hypothesized to be non-mobile in the majority of the cases.

Moreover, the contribution of these two GIs towards metal susceptibility phenotypes was addressed by generating deletion mutants. Susceptibility data of both G08 and G62 mutants respectively of ATCC 17978 and A424 showed a significant, but minor decrease in the MIC for zinc (ZnSO_4_), cobalt (CoCl_2_), cadmium (CdCl_2_), and nickel (NiSO_4_), whereas the MIC remained unchanged for copper (CuSO_4_), iron (FeSO4), and arsenic (As_2_O_3_). These GI deletion mutants showed slight phenotypic changes as compared to their wild type counterparts, and their tolerance to the rest of the metals could be attributed the presence of other chromosomal efflux transports. Putative efflux pumps for copper and zinc have been previously analyzed in *A. baumannii* ATCC 17978 by TransAAP [44]. Thirteen efflux systems were identified that belong to either the CDF family, P-type ATPase family, CorA metal ion transporter family, HME family of RND transporters, or CopB- type family of Cu exporters. Transcriptional data by qPCR revealed that some of these putative efflux genes were induced by addition of either or both zinc and copper [16]. The MIC/MBC data of broth microdilution were identical in both A424 and ATCC 17978 wild-type and mutants for copper, cobalt, iron, and nickel. This observation was partially explained by the presence of *cnrCBA* mediating resistance to cobalt-nickel, as well as *czcCBA* genes which are cobalt-zinc-cadmium resistance determinants in this bacterial strain [45,46]. Similar iron susceptibility data in both wild-type and mutants of ATCC 17978 and A424 could be due to the presence of putatively non-functional FeoB in ATCC 17978, and the tolerance could be attributed to another iron efflux transporter. The additive value of metal resistance carried on mobile elements, for example the copper resistance conferred by the *Staphylococcus aureus* COMER element in USA3000, still confers the strain’s increased resistance to copper-related macrophage killing, and showed significant higher virulence, despite the weak phenotypes detected in vitro [47].

Collectively, this work reveals that metal resistance GIs in *A. baumannii* are of clinical significance as they confer metal resistance phenotypes, and their mobility could be demonstrated by the integrase assays. This issue can raise concern as these metal-resistance GIs could be readily transferred among strains (and patients) in clinical settings, and could be viewed as vehicles disseminating resistance as well as other potential virulence genes. The use of sub-lethal doses of antimicrobials and metal-containing compounds not only accelerate their resistance, but could also potentiate their virulence and spread in hospital environments.

## Figures and Tables

**Table 1:** List of *A. baumannii* strains.

Strain	ST	Relevant Characteristics	Reference
A424	1	Clinical isolate from Croatia	[11]
A424 ΔG08	1	*G08::aacC1*	This study
A424 pWSK129-WHG08	1	Complemented ΔG08 strain	This study
AYE	1	Epidemic MDR type strain, France	[10]
AB0057	1	MDR type strain	[23]
ATCC 17978	437	Reference strain	[17]
ATCC 17978 ΔG62	437	*G62::aacC1*	This study
ATCC 17978 pWSK129-WHG62	437	Complemented ΔG62 strain	This study
KR3831	1	Clinical isolate from SQUH, Oman	This study

ST = Sequence type [24].

**Table 2:** Metal susceptibility testing wild type (wt) and GI mutants.

	ATCC 17978	ATCC 17978 ΔG62	A424	A424 ΔG08
ZnSO_4_	4 *	2	0.5	0.5
CuSO_4_	8	8	8	8
CdCl_2_	4	2	2	2
MnSO_4_	10	10	1	0.5
FeSO_4_	4	4	4	4
CoBr_2_	4	2	4	4
NiSO_4_	10	5	10	10
As_2_O_3_	4	4	4	4

* MIC in μg/mL.

## Supplementary Materials

The following are available online at http://www.mdpi.com/2073-4425/9/7/366/s1, Table S1: List of primers, Figure S1: Standard curves for the qPCR.
