# Supplementary material for: Integrase-Controlled Excision of Metal-Resistance Genomic Islands in Acinetobacter baumannii
Source: Genes (Basel). 2018 Jul 20;9(7):366. doi: 10.3390/genes9070366 (PMC6070778; doi:10.3390/genes9070366)
Supplement: Supplementary file 1 [file genes-09-00366-s001.docx]

**Table S1.** List of primers.

| Name | Sequence (forward) | Sequence (Reverse) | comments |
| --- | --- | --- | --- |
| alx_UG08 | CTGCAGGTGATAGGCTTCACGGCCAA | TGGTACTTCAAGATCCCCAATTCGTAATAAAACCAT | UF of G08 with restriction site for allelic exchange |
| Alx_DG08 | GCTTTTGAAGCTAATTCGTAATGGCTGCATAAA | GTCGACTGCGGGCTATTTTTGGTAAGCTGG | DF of G08 with restriction site for allelic exchange |
| Gm | CGAATTAGCTTCAAAAGCGCTCTGA | CGAATTGGGGATCTTGAAGTTCCT | Amplification of FRT-gentamicin-FRT cassette |
| lam_F8 | CATGTACAACAAAGTGAGTACAACCTGTTGCT | AGCTGGCTCGGCATTAGGGATTTTTGC | SOE amplicon of UF+*aacC1* cassette +DF of G08 |
| 16S | ACTCCTACGGGNGGCNGCA | GTATTACCGCNNCTGCTGGCAC | 16S gene for qPCR |
| G08-exc | ATGTTTAGCTGCGTAAATCG | TTTGGTTGCTGGTGTGGACAA | G08 junction by qPCR |
| G08-circ | TTCAGAATAAGCTGGATAGC | TAAGCGTTAGGTGTTGGGTC | G08 excision by qPCR |
| G62-exc | AACAAATATGAACTAATTGC | TTGTTGAAGACTATTAATG | G62 junction by qPCR |
| G62-circ | CAAGACAACCTGCTTTTACG | CGATATGGTTTGTCTTGTGG | G62 excision by qPCR |
| G08-int-clon | CGAGAGCTCAATAAATGTTAAAAGATACC | GTGTGTCGACTTAAGTGACCTCCTCATGC | Cloning of G08int |
| G62_int_clon | AAAAGCTTATTGAGTATGGCAAAGCACCG | CTTTAACTGCATATGTAGGATGCCGACTG | Cloning of G62int |
| A424-DCO | TCGCCATAACCTATGCTGGC | CAACAACAGAAGCGGCAGCC | DCO G08 mutant check |
| 17978-DCO | GTCACGATTCACGAAGATGA | GCTGCCTGACGCATAATCAC | DCO G62 mutant check |
| AB57-int | ATGAAGTTGGGAGCGAATACGG | CCAAGATTCGGCACACCTTTC | Screening G08 int |
| 17978-int | CATCGATAA CGGTCTGGCATG | AAGTACCGGTGAAACGACAGC | Screening G62 int |
| PR3136/PR3137 | CGGGATCCAATGCAGGCAAGTTGATTCC | CGGGATCCCGTTCGGGTCTTTCATGTCT | Ori from pWH1266 for cloning into pWSK129 |
| LF-RS-G08 | CTGCAGGTGATAGGCTTCACGGCCAA | GTACTTCAAGATCCCCAATTCGTAATAAAACCATTG | LF G08 Mutant validation colony PCR |
| RF-RS-G08 | GCTTTTGAAGCTAATTCGTAATGGCTGCATAAA | GTCGACTGCGGGCTATTTTTGGTAAGCTGG | RF G08 Mutant validation colony PCR |
| LF-RS-G62 | CGAATTGGGGATCTTGAAGTACCTATTCCG | CGAATTAGCTTCAAAAGCGCTCTGAAGTTCC | LF G62 Mutant validation colony PCR |
| RF-RS-G62 | CATGTACAACAAAGTGAGTACAACCTGTTGCT | AGCTGGCTCGGCATTAGGGATTTTTGC | RF G62 Mutant validation colony PCR |
| Gm-S | CATTAAACTGCTCATTCCAG | TAAGGCATTTGGCCGCATTG | *aacC1* screening in SCO of allelic exchange |
| G08-S | TTAAAACCACAAGACAAACCTCAGCCC | CCATTGAAGCATAGAGCGTCCCAGTG | SCO screening in allelic exchange of G08 |
| G62-S | TCACCGAGGACTCCTTCTTC | GGCGCAATACGTCTGATCTC | SCO screening in allelic exchange of G62 |
| LF-08 | TGATGTTTCTATCGAATTAATGTCATG | AACTTAATCTTTAAAGTTATT GCTA | Cloning of UF in mini islands of G08 in pUC18 |
| RF-08 | AATAACTTTAAAGATTAAGTTACTG | ATCCTCTTTTAAAGTTTTGACTA | Cloning of DF in mini islands of G08 in pUC18 |
| LF-08 | AGTAGTCGATGATGAAATAGTTGATGC | CGCGTTAATTCCTGCTATGGTCC | Cloning of LF in mini islands of G62 in pUC18 |
| RF-62 | AAAAGCTTATTGAGTATGGCACTGC | CCTTTAACTGCATATGTAGGCCGGTA | Cloning of RF in mini islands of G62 in pUC18 |
| F/RG08int | CATTAATCTTTAAAGTTATTGCATC | GATAGTAAAAAGTCTTAATACTGACT | Cloning of G08 integrase gene into pUC18 |
| F/RG62int | CGAGTCGACAATAAATGTTAAAAGATACC | GTGTGGATCCTTAAGTGACCTCCTCATGC | Cloning of G62 integrase gene into pUC18 |
| EXC8-F/R | TGTTCTTGTCCTTTACTGTG | GAAGAGGGTAAATCTTCTCC | Detection of circular forms of G08 from chromosome |
| JUNC8-F/R | TAACGGTCAAAGGTAACGTG | ATCAATTCCACTACAGTTG | G08 target Junction from chromosome |
| EXC62-F/R | TGTTGTAGGAATATTACTCG | ATGCATATTCTTTTTACACC | Detection of circular forms of G62 from chromosome |
| JUNC62-F/R | AAATCGGAAGAACAGCCAGC | ATGAGAAGTCGGTAAGGGC | G62 target Junction from chromosome |
| PS-F/R | TTACCCAAGACTTCGCCTCT | CCCGATAACTCCATTCTTCG | Loss of pJTOOL-3 backbone |
| 2LF-08/2RR-08 | AATTGGGGATCTTGAAGTTCCT | GATGGCGTAAATCGTGGTA | Flank1 *aacC1* in mutant alleles of G08 |
| 1LF-08/1RR-08 | CAACTTTAGCGATTTCTGG | CTTTAGCAAACATGACCTACC | Flank2 *aacC1* in mutant alleles of G08 |
| 2LF-62/2RR-62 | TACACCCGGGTTAATCGT | GCGCTTCAAAATCTGATGTA | Flank1 *aacC1* in mutant alleles of G62 |
| 1LF-62/1RR-62 | GCGTATATTTTGTTTCCATTC | GACCTTTCTTATCACAACGA | Flank2 *aacC1* in mutant alleles of G62 |

**Figure S1.** Standard curves for the qPCR. The log dilutions of DNA are plotted versus the cycle threshold (Ct) values. The equation of the regression line and its R^2^ values are displayed in the upper right corner of each graph. For each qPCR assay a standard curve was generated using four 5-fold dilutions of gDNA. All these assays were conducted in *A. baumannii* background (primer pairs used in these curves are: (A)16S-F/R, (B) gyrB-F/R (C) G08-EXC F/R, (D) G08-JUN F/R, (E) G62-JUN F/R, (E) G62-EXC F/R (Listed in table S1).
